# Docking Studies, Synthesis, Characterization and Evaluation of Their Antioxidant and Cytotoxic Activities of Some Novel Isoxazole-Substituted 9-Anilinoacridine Derivatives

**DOI:** 10.1100/2012/165258

**Published:** 2012-04-19

**Authors:** R. Kalirajan, M. H. Mohammed Rafick, S. Sankar, S. Jubie

**Affiliations:** Department of Pharmaceutical Chemistry, JSS College of Pharmacy, JSS University, Tamilnadu Ootacamund 643001, India

## Abstract

A convenient synthesis of novel isoxazole-substituted 9-anilinoacridine derivatives **5a–j** was reported. The compounds were confirmed by physical and analytical data and screened for *in vitro* antioxidant activity by DPPH method, reducing power assay and total antioxidant capacity method. The cytotoxic activity of the compounds was also studied in HEp-2 cell line. The docking studies of the synthesized compounds were performed towards the key nucleoside dsDNA by using AutoDock vina 4.0 programme. All the isoxazole-substituted compounds have significant activities.

## 1. Introduction

Acridine derivatives are used in medicine and have enormous potential as pharmaceutical agents due to their biological activities such as antimicrobial [1], antioxidant [2], anticancer [3–5], antimalarial [6], anti-inflammatory [7], analgesic [8], antileishmanial [9], and so forth. The chemical modification of acridines such as the introduction of different substitutions or heterocyclic rings were allowed expansion of research on the structure activity relationship to afford new insight into molecular interactions at the receptor level [10]. In fact, it is well established that slight structural modification on 9-anilinoacridines may bring various pharmacological effects. Similarly isoxazoles [11–13] also have various biological activities like as antimicrobial, anticancer, and so forth. In order to look for some new compounds with interesting biological properties, we would like to synthesize some novel isoxazole-substituted 9-anilinoacridine derivatives.

## 2. Experimental

**Scheme 1:**
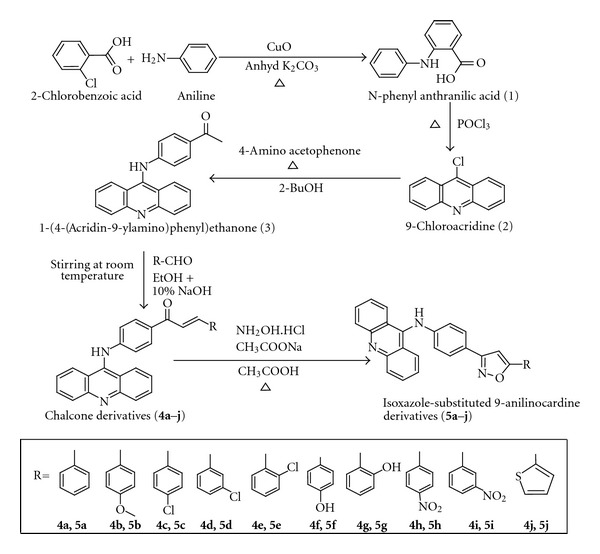


All the organic solvents used were of high purity unless otherwise stated. The reactions were monitored by TLC on silica gel thin layer plates. Elemental analysis (C, H, N; Elementar Vario EL III Instrument), melting points (in glass capillary tubes on a Veego VMP-1 Apparatus), IR spectra (Shimadzu 8400 FT-IR spectrometer), ^1^H NMR and ^13^C NMR (Bruker A VIII 500 MHz Spectrometer) spectra have been recorded. Mass spectra of the final compounds were recorded on a JEOL GC mate Mass Spectrometer. The synthetic procedure was explained in the Scheme 1.

### 2.1. Synthesis of 1-[4-(Acridin-9-Ylamino)Phenyl]Ethanone 3

In a 250 mL round-bottomed flask 4.06 g (0.03 mole) of 4-aminoacetophenone was refluxed with 5.4528 g (0.0256 mole) of 9-chloroacridine in 80 mL of 2-butanol for 3 h. After completion of reaction the reaction mixture was allowed to cool to room temperature then it was poured into 150 mL of ice water. A precipitate formed was filtered by suction, washed with water, dried, and recrystallized from ethanol.

### 2.2. General Procedure for Synthesis of Chalcones **4a–j**

The chalcones were synthesized by using general Claisen-Schimdt condensation. In a 100 mL flat-bottomed flask 25 mL of the 10% sodium hydroxide and 25 mL of ethanol were taken with a magnetic stirring bar, it was placed on the magnetic stirrer, and the stirring dial was adjusted to get a nice, even stirring action. To this 0.0115 mole of corresponding aldehyde was added, then 2.9952 g (0.0096 mole) of 1-[4-(acridin-9-ylamino)phenyl]ethanone was added at the last. The solution was allowed to stir for 8 h at room temperature. After completion of the reaction 100 mL of water was added, formed precipitate was filtered and washed three times with 50 mL of water each time to remove sodium hydroxide, dried, and crystallized from ethanol. All the compounds are characterized and confirmed by IR, NMR, and mass spectroscopy. All the values are agreed with the synthesized compounds.

The yield of compounds is between 60 and 85%; IR (KBr, *ν*, cm^−1^) for compound **4a**: 3269 (N–H), 3057-3000 (Ar C–H), 1647 (*α*,*β*-unsat. C=O), 1604 & 1516 (Ar C=C), 1226 (C–N), 759 (Ar C–H); ms : *m/z* 400.16 (M^+^); ^1^H NMR (in ppm): 6.65–8.02 (16H, m, ArH), 7.90 and 7.56 (2H, s, *α*,*β*-unsaturated), 11.21 (1H, s, NH); ^13^C NMR (in ppm): 183.4, 153.8, 149.8, 142.8, 140.5, 136.1, 135.4, 131.7, 131.3, 130.4, 129.3, 129.8, 128.9, 127.8, 126.7, 126.7, 121.9, 119.3, 115.7.

### 2.3. General Procedure for Synthesis of Isoxazole-Substituted 9-Anilinoacridines **5a–j**

In a 25 mL beaker, 2.5 g (0.02 mol) anhydrous sodium acetate was dissolved in minimum amount of hot glacial acetic acid (10 mL) and it was added to a solution of 0.81 g (0.01 mol) hydroxyl amine hydrochloride in absolute ethanol (10 mL). A solution of 0.01 mol of the corresponding chalcones **4a**–**j** in 10 mL of absolute ethanol and the above solution were refluxed for 6 hours in oil bath. The reaction was monitored by TLC (pet ether: ethyl acetate 3 : 2). After completion of the reaction excess solvent was concentrated in vacuo at reduced pressure, residual solution obtained was cooled to room temperature, poured into ice cold water, neutralized with sodium hydroxide, and washed well with water to remove unreacted hydroxyl amine hydrochloride and excess of sodium hydroxide. Dried crude product yield was noted and recrystallized from ethanol. All the compounds are characterized and confirmed by IR, NMR and mass spectroscopy. All the values are agreed with the synthesized compounds.

This compound is obtained as a yellow powder; m.p.: 170–172°C; Yield: 56%; Anal. calc. for C_28_H_19_N_3_O: C, 81.34; H, 4.63; N, 10.16; O, 3.87. Found: C, 81.21; H, 4.42; N, 10.1; IR (KBr, *ν*, cm^−1^): 3340 (N–H), 2993 (Ar st C–H), 1600 & 1473 (Ar C=C), 1157 (Ar C=N), 1226 (C–O), 742 (Ar C–H); MS: *m/z* 413.15 (M^+^); ^1^H NMR (in ppm): 6.85–8.94 (16H, m, ArH), 9.95 (1H, s, CH of isoxazole), 11.10 (1H, s, 9-NH); ^13^C NMR (in ppm): 98.4 (C of isoxazole), 114.8–143.1 (27 aromatic carbons).

### 2.4. Molecular Docking

**Figure 1:**
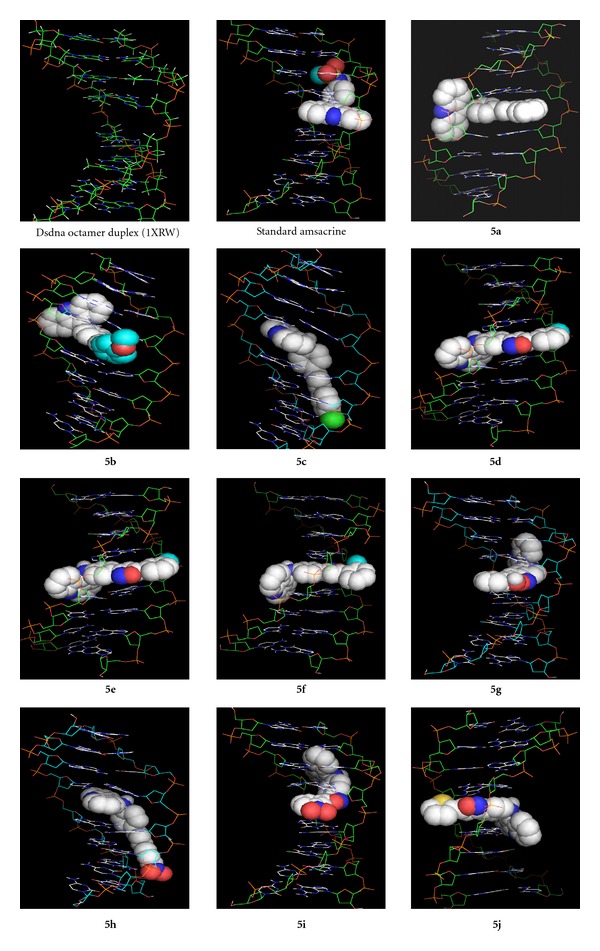
Best affinity mode of docked compounds.

The computer-simulated automated docking studies were performed using the widely distributed molecular docking software, AutoDock Vina 4.0. The proteins were extracted from protein database at the NCBI, that is, dsDNA octamer duplex (1XRW). The designed 9-anilinoacridine analogues were taken for prediction of 3D structure by using Cambridge software. The energy was minimized for flexible docking using Argus Lab, for the docking of ligands to protein active sites and for estimating the binding affinities of docked compounds by an advanced molecular docking program AutoDock Vina, version 4.0. Predicting the binding affinity and rank-ordering ligands in database screens was implemented by modified and expanded version of the ChemScore18 scoring functions, for use. Using standard precision (SP) mode of PyMOL software, docking studies were performed on planned compounds for synthesis. The structures of the compound and enzymes are shown in Figure 1.

## 3. Biological Evaluation

Acridine derivatives possess a diverse range of pharmacological activities [14–16]. Hence all the chalcone and isoxazole-substituted 9-anilino acridine derivatives **4a–j, 5a–j** were screened for antioxidant activity and short-term *in vitro *antitumor activity against Daltons Lymphoma Ascites (DLAs) cells. Many of the synthesized final compounds **5a–j** have significant activities.

### 3.1. Antioxidant Activity

#### 3.1.1. DPPH Assay [17]

The assay was carried out in a 96-well microtitre plate. To 200 *μ*L of DPPH solution, 10 *μ*L of each of the synthesized or standard solution was added separately in wells of the microtitre plate. The final concentration of the synthesized compounds and standard solutions used were 1000, 500, 250, 125, 62.5, 31.25, and 15.625, 7.812 *μ*g/mL. The plates were incubated at 37°C for 30 min and the absorbance of each solution was measured at 490 nm, using ELISA reader. The percentage inhibition was calculated. IC_50_, which is the concentration of the sample required to scavenge 50% of free radicals.

#### 3.1.2. Reducing Power Assay [18]

10 mL of each of the synthesized or standard solution was added separately in 1.0 mL of deionized water that were mixed with phosphate buffer (2.5 mL) and potassium ferri cyanide (2.5 mL). The mixture was incubated at 50°C for 20 min. Aliquots of trichloroacetic acid (2.5 mL) were added to the mixture, which was then centrifuged at 3000 rpm for 10 min whenever necessary. The upper layer of solution (2.5 mL) was mixed with distilled water (2.5 mL) and a freshly prepared ferric chloride solution (0.5 mL). The absorbance was measured at 700 nm. Ascorbic acid at various concentrations was used as a reference standard. Phosphate buffer (pH 6.6) was used as blank solution. The absorbance of the compounds taken was expressed as mean ± standard deviation. Increased absorbance of the reaction mixture indicates increase in reducing power.

#### 3.1.3. Antioxidant Potential Assay by Phosphomolybdenum Method [19]

The antioxidant power of the compounds has been assessed with the phosphomolybdenum reduction assay according to Armatu et al. The assay is based on the reduction of Mo(VI)-Mo(V) by the compounds and subsequent formation of a green phosphate—Mo(V) complex at acid pH. Various concentrations compounds were combined with 3 mL of reagent solution (0.6 M sulfuric acid, 28 mM sodium phosphate, and 4 mM ammonium molybdate). The tubes containing the reaction solution were incubated at 95°C for 90 min. Then the absorbance of the solution was measured at 695 nm using spectrophotometer against blank after cooling to room temperature. DMSO (0.3 mL) in the place of compounds was used as the blank. For reference, the various concentrations of ascorbic acid have been used.

#### 3.1.4. Short-Term Study for *In Vitro* Antitumor Activity [20]

Cells were collected, counted, and adjusted to 1 × 10^6^ cells/mL. The drug dilutions were made with phosphate buffer saline, and the drug dilutions were further adjusted to required concentrations. The drug dilutions were then added to the Daltons Lymphoma Ascites (DLAs) cells and incubated at 37°C for 3 hours. At the end of 3 hours, trypan blue dye exclusion test was performed, and percentage viability and percentage cytotoxicity were calculated.

## 4. Results and Discussion

For *in vitro* antioxidant activity, all the synthesized chalcone and isoxazole-substituted 9-anilino acridines were screened by DPPH assay, reducing power assay, and TAC almost all the isoxazole-substituted derivatives have shown antioxidant activity, among these compounds **5a, 5c, 5d, 5f, 5i, **and** 5j **having more potent antioxidant activity when compared to standard ascorbic acid. The IC_50_ values, which is the concentration of the sample required to increase 50% of reducing capacity of the compounds, are shown in Table 1.

The synthesized final compounds **5a–j** were subjected to short-term study for *in vitro *cytotoxic activity against Daltons Lymphoma Ascites (DLA) cells. All the isoxazole substituted derivatives were shown significant cytotoxicity and the compound **5i** has more potent cytotoxic activity. The results were shown in Table 2.

For the docking of ligands to protein active sites and for estimating the binding affinities of docked compounds, an advanced molecular docking program, AutoDock Vina version 4.0, was used in this study. The designed analogues are docked towards the dsDNA octamer duplex in order to ascertain their anticancer activity. The analogues show best fit Root Mean Square Difference (rmsd) value of 0.000.

## 5. Conclusion

A series of novel isoxazole-substituted 9-anilino acridine derivatives have been synthesized. The synthesized compounds have significant antioxidant activity when compared with standard ascorbic acid and also showed significant cytotoxic activity by* in vitro* anticancer activity when compared to the control. The molecular docking studies show a good correlation between their biological activities screened and autodock binding free energy. In particular compound **5i** shows significant anticancer activity. Compound **5i **have potent cytotoxic activity and is likely to be useful as drugs after further refinement. These derivatives will encourage helping to design future anticancer agents with therapeutic potentials.

## Figures and Tables

**Table 1:** Antioxidant activity of synthesized compounds **4a–j** and **5a–j** by DPPH method, reducing power assay and total antioxidant capacity method.

S. No	Cpd. Code	IC_50_ values for DPPH method (*μ*g/mL)	IC_50_ values for reducing power assay (*μ*g/mL)	IC_50_ values for TAC (*μ*g/mL)
(1)	**4a**	>1000	>500	>500
(2)	**4b**	>1000	>500	>500
(3)	**4c**	>1000	>500	>500
(4)	**4d**	>1000	>500	>500
(5)	**4e**	>1000	>500	>500
(6)	**4f**	>1000	>500	>500
(7)	**4g**	>1000	>500	>500
(8)	**4h**	>1000	>500	>500
(9)	**4i**	>1000	>500	>500
(10)	**4j**	>1000	>500	>500
(11)	**5a**	18.30 ± 0.4583	34.17 ± 0.5783	89.60 ± 0.7767
(12)	**5b**	50.23 ± 1.048	68.27 ± 0.5608	122.0 ± 1.044
(13)	**5c**	17.53 ± 0.088	22.13 ± 0.5044	92.80 ± 0.9074
(14)	**5d**	14.20 ± 0.1528	26.43 ± 0.5696	81.83 ± 0.2404
(15)	**5e**	33.20 ± 1.323	43.37 ± 0.5925	111.8 ± 0.8819
(16)	**5f**	12.67 ± 0.1202	29.50 ± 0.8718	91.43 ± 0.2404
(17)	**5g**	27.00 ± 0.8660	47.03 ± 1.035	132.3 ± 0.8090
(18)	**5h**	28.20 ± 0.3464	37.30 ± 0.4619	75.43 ± 0.2404
(19)	**5i**	13.63 ± 0.1764	19.23 ± 0.4667	95.43 ± 0.2404
(20)	**5j**	16.30 ± 0.5568	20.53 ± 1.004	65.97 ± 0.5207
(21)	Standard ascorbic acid	18.60 ± 1.039	24.77 ± 0.2404	86.17 ± 0.5783

**Table 2:** Short term *in vitro *anti-tumor activity of synthesized compounds against Daltons Lymphoma Ascites (DLA) cells.

S. No	Compound	Concentration (*μ*g/mL)	% viability	% cytotoxicity	Cyto_50_ (*μ*g/mL)
(1)	**5a**	1000	35.83	65.83	679.08
		500	58.02	41.98	
		250	75.58	24.42	

(2)	**5b**	1000	40.56	59.44	853.95
		500	65.29	34.71	
		250	75.54	24.46	

(3)	**5c**	1000	35.23	64.77	788.77
		500	65.17	34.83	
		250	78.66	21.34	

(4)	**5d**	1000	43.33	56.57	518.29
		500	50.57	49.43	
		250	70.27	29.73	

(5)	**5e**	1000	45.78	54.22	947.96
		500	77.27	22.73	
		250	85.71	14.29	

(6)	**5f**	1000	46.87	53.13	633.71
		500	59.35	40.65	
		250	88.26	11.74	

(7)	**5g**	1000	46.38	53.62	938.70
		500	69.98	30.31	
		250	79.67	20.33	

(8)	**5h**	1000	32.50	67.50	539.81
		500	53.33	46.67	
		250	81.70	18.30	

(9)	**5i**	1000	38.37	61.63	311.75
		500	48.42	51.58	
		250	58.33	41.67	

(10)	**5j**	1000	47.11	52.89	930.27
		500	71.51	28.49	
		250	89.32	10.68	
